# Predicting Sleep Quality through Biofeedback: A Machine Learning Approach Using Heart Rate Variability and Skin Temperature

**DOI:** 10.3390/clockssleep6030023

**Published:** 2024-07-23

**Authors:** Andrea Di Credico, David Perpetuini, Pascal Izzicupo, Giulia Gaggi, Nicola Mammarella, Alberto Di Domenico, Rocco Palumbo, Pasquale La Malva, Daniela Cardone, Arcangelo Merla, Barbara Ghinassi, Angela Di Baldassarre

**Affiliations:** 1 Department of Medicine and Aging Sciences, “G. D’Annunzio” University of Chieti-Pescara, 66100 Chieti, Italy; pascal.izzicupo@unich.it (P.I.); giulia.gaggi@unich.it (G.G.); b.ghinassi@unich.it (B.G.); a.dibaldassarre@unich.it (A.D.B.); 2 UdA-TechLab, “G. D’Annunzio” University of Chieti-Pescara, 66100 Chieti, Italy; arcangelo.merla@unich.it; 3 Department of Engineering and Geology, “G. D’Annunzio” University of Chieti-Pescara, 65127 Pescara, Italy; david.perpetuini@unich.it (D.P.); d.cardone@unich.it (D.C.); 4 Department of Psychological, Health and Territorial Sciences, “G. D’Annunzio” University of Chieti-Pescara, 66100 Chieti, Italy; nicola.mammarella@unich.it (N.M.); alberto.didomenico@unich.it (A.D.D.); rocco.palumbo@unich.it (R.P.); pasquale.lamalva@unich.it (P.L.M.)

**Keywords:** sleep quality, wearable sensors, contactless sensors, heart rate variability, skin temperature, infrared thermography, machine learning

## Abstract

Sleep quality (SQ) is a crucial aspect of overall health. Poor sleep quality may cause cognitive impairment, mood disturbances, and an increased risk of chronic diseases. Therefore, assessing sleep quality helps identify individuals at risk and develop effective interventions. SQ has been demonstrated to affect heart rate variability (HRV) and skin temperature even during wakefulness. In this perspective, using wearables and contactless technologies to continuously monitor HR and skin temperature is highly suited for assessing objective SQ. However, studies modeling the relationship linking HRV and skin temperature metrics evaluated during wakefulness to predict SQ are lacking. This study aims to develop machine learning models based on HRV and skin temperature that estimate SQ as assessed by the Pittsburgh Sleep Quality Index (PSQI). HRV was measured with a wearable sensor, and facial skin temperature was measured by infrared thermal imaging. Classification models based on unimodal and multimodal HRV and skin temperature were developed. A Support Vector Machine applied to multimodal HRV and skin temperature delivered the best classification accuracy, 83.4%. This study can pave the way for the employment of wearable and contactless technologies to monitor SQ for ergonomic applications. The proposed method significantly advances the field by achieving a higher classification accuracy than existing state-of-the-art methods. Our multimodal approach leverages the synergistic effects of HRV and skin temperature metrics, thus providing a more comprehensive assessment of SQ. Quantitative performance indicators, such as the 83.4% classification accuracy, underscore the robustness and potential of our method in accurately predicting sleep quality using non-intrusive measurements taken during wakefulness.

## 1. Introduction

Sleep is part of the circadian rhythm and is characterized by sequences of stages with related autonomous nervous system (ANS) functions [1]. It is a complex physiological process that covers nearly one-third of the lifespan and plays a relevant role in the consolidation of memories, learning, physical development and fitness maintenance, emotion regulation, and quality of life [2]. Sustained deprivation of sleep leads to a decrease in the immune system’s efficiency and increases the risk of cardiovascular pathologies, hypertension, obesity, metabolic deregulation, and diabetes [3]. Poor SQ is associated with a large annual economic loss due to the reduction in workplace productivity, with an estimated value ranging from $299 billion to $433 billion by the year 2020 in the United States [4]. It has been reported that almost fifty percent of older adults experience impaired SQ, and it has been estimated that the prevalence is lower in healthy adults; therefore, SQ may be regarded as an early indicator of cognitive decline in midlife [5]. Hence, it is expected that SQ examination will become a major relevant analysis for the medical diagnosis. SQ is likely a multifaceted construct that would be difficult to characterize by any single measure, requiring a multimodal approach investigating physiological changes induced by poor SQ.

Both subjective and objective methodologies can be used to assess SQ. Among the subjective methods, the sleep diary is the most extensively used [6], as it requires the individual to record morning estimates of their sleep pattern parameters. However, its success relies heavily on daily (prospective) recordings as soon as individuals wake up in the morning, which may be a challenging task for older individuals to consistently remember to perform. In contrast, retrospective self-report measures, such as questionnaires, can be widely used in both routine care and clinical trials due to their low cost, high patient compliance, ease of administration, and potentiality to be administered to a variety of populations via the Internet [7]. The Pittsburgh Sleep Quality Index (PSQI) is a self-reported survey that evaluates SQ and disruptions within the preceding four weeks [8]. The assessment tool consists of a total of 18 items that are categorized into seven distinct components, namely, subjective SQ, sleep latency, sleep duration, habitual sleep efficiency, sleep disturbances, use of sleeping medication, and daytime dysfunction. A cut-off score of 5 was defined to distinguish between individuals with good and poor SQ; elevated scores (i.e., >5) are indicative of suboptimal subjective SQ [8].

Concerning objective methodologies to assess SQ, polysomnography (PSG) is an objective methodology with a high degree of reliability for obtaining data on sleep parameters [9]. However, objective methods are generally costly and time-consuming [9]. Notably, low-cost wearable sensors able to record the wearer’s heart rate (HR) are currently used to assess SQ in a non-invasive manner [10]. Specifically, wearable devices can capture several physiological signals useful for SQ assessment, such as HRV, electrodermal activity, body movement, skin temperature, respiratory signals, and brain activity. Exploiting machine learning (ML) frameworks, it is possible to deliver generalizable classifications of sleep quality from the recordings of these physiological signals [11]. SQ is correlated with HRV metrics during sleep, highlighting the close interconnection between sleep and ANS activity. HRV metrics during sleep stages show distinct associations with clinical indicators of metabolic function, indicating the influence of sleep on ANS and metabolic regulation. HRV analysis during sleep provides a model to investigate ANS activity and its fluctuations caused by intrinsic factors, such as circadian rhythm, without the confounding influence of daytime activities. Significant differences in HRV metrics among different sleep stages indicate the dominance of sympathetic nervous system (SNS) activity during unstable sleep and the dominance of parasympathetic nervous system (PNS) activity during stable sleep. Overall, HRV analysis during sleep stages allows the identification of distinct ANS function patterns and their associations with metabolic function, providing valuable insights into the regulation of ANS function and metabolic processes during sleep [12]. Importantly, several studies evaluated the relationship between HRV and SQ during resting wakefulness, demonstrating an influence of the quality of sleep on HRV metrics even during the waking state. For instance, Gouin et al. [13] found that greater HRV during resting wakefulness is associated with better sleep efficiency as measured with sleep diaries over one week in young adults. The results suggest that HRV during a short resting period is an independent index of sleep efficiency and could be used as a clinical biomarker of sleep quality. In addition, Van den Berg et al. [14] found that HR changed significantly sooner when subjects were sleep-deprived than when they were rested during a monotonous attention task lasting 120 min.

From this perspective, it is worth highlighting that changes in HRV can produce modifications in the peripheral circulation that can be easily assessed through infrared thermography (IRT). IRT is a technique used to capture the infrared radiation emitted by an object in a contactless manner, allowing us to estimate the superficial temperature of the object. Its effectiveness as a complementary tool alongside other diagnostic methods has been demonstrated in several applications in the biomedical field, such as cancer detection [15,16], vascular disorder evaluation [17], musculoskeletal injury monitoring [18], and inflammatory state identification [19]. IRT has been used to assess changes in the breathing rate through the temperature modulations of the regions around the nostrils and the mouth [20] during the sleepy state. Notably, thus far, IRT has not been used to assess the quality of sleep during the awake state. However, facial skin temperature oscillations evaluated through IRT have been demonstrated to be related to HRV metrics [21], hence suggesting that skin temperature modulations during the awake state could be influenced by the quality of sleep as the HRV is. Importantly, HRV metrics and skin temperature are not correlated per se, but skin temperature is related to the superficial microcirculation, which, in turn, is related to blood flow and volume, which are dependent on the heart rate, making it possible to develop models able to estimate HRV parameters from features extracted from skin temperature oscillations [22]. Thus, using HRV and IRT together could improve their sensitivity in predicting SQ, providing an accurate objective estimate of such a physiological state.

The objective of this study was to estimate the SQ through ML approaches applied to both HRV and IRT signals using the PSQI as a gold standard. Specifically, cross-validated classifiers were employed to predict PSQI scores and to provide a two-class classification (i.e., good and poor SQ, using PSQI = 5 as the cut-off score) respectively. The remainder of this document is organized as follows: in the following section, the study design, the participant recruitment, the data collection procedures, and the methods used for HRV and IRT measurement are described. Additionally, this article outlines the machine learning algorithms employed and the validation procedures used in the study. Then, the performance of our ML algorithm is provided. Then, the strengths, limitations, and key findings of the study are outlined.

## 2. Results

### 2.1. Experimental Design

**Figure 1:**
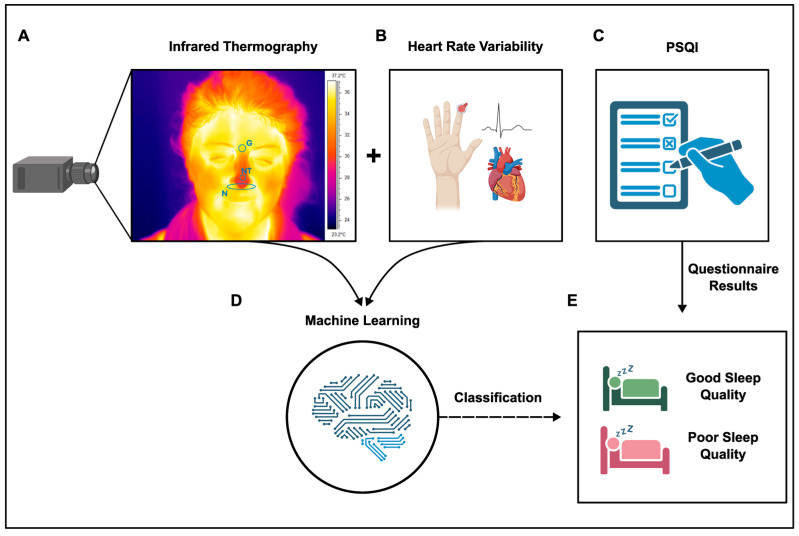
Schematics of the experimental procedures. (**A**) Thermogram of a representative participant showing the ROIs’ positions covering the glabella (G), nose tip (NT), and nostrils (N); skin temperature data and (**B**) HRV metrics were the two objective physiological signals obtained. Additionally, (**C**) sleep quality was subjectively assessed using the PSQI. (**D**) Machine learning using thermic and HRV data separately or in combination was used to estimate and (**E**) classify sleep categories. The image was created using BioRender.com.

IRT and HRV were measured at rest for 5 min in our experimental group of administrative employees. Data regarding subjective sleep quality were also obtained using the PSQI. ML was implemented, and objective IRT and HRV data were used as input features to classify “good and poor sleep” groups, based on PSQI data. Figure 1 displays the experimental design and procedure adopted in this study.

### 2.2. Machine Learning Accurately Classifies Sleep Quality Using HRV and Skin Temperature

The features identified by the feature selection procedure for the HRV were the mean, maximum, and standard deviation of the HR; very low frequency (VLF); low frequency (LF); ratio between low frequency and high frequency (LF/HF); and SD2/SD1. Concerning the IRT, the features selected were the delta of temperature of the glabella, nose tip, and nostrils; the skewness of the temperature of the nose tip; the SampEn of the nose tip; the PSD of the respiratory band of the glabella; and the PSD of the myogenic band of the nose tip.

In selecting classifiers for a classification problem, it is essential to utilize a diverse set of models to ensure robust and comprehensive analysis. The chosen classifiers—Decision Tree (DT), Support Vector Machine (SVM) with a linear kernel, k-nearest neighbor (KNN), Ensemble (ENS), and neural network (NN)—each bring distinct advantages. For instance, Decision Trees effectively capture non-linear relationships between features and target variables. Support Vector Machines with a linear kernel are particularly effective in high-dimensional spaces. The k-nearest neighbor algorithm is noted for its simplicity and intuitiveness, making predictions based on the majority class among the nearest neighbors without assuming any underlying data distribution. Ensemble methods combine the strengths of multiple base models to improve overall performance. Neural networks are capable of learning complex and non-linear patterns in the data, making them ideal for problems where such patterns are present. This selection ensures a comprehensive evaluation of the classification problem, leveraging the unique strengths of each classifier. However, it should be highlighted that evaluating all these models allowed a benchmarking procedure. Specifically, the best performances of the models were considered in terms of TPR, TNR, and accuracy. Here, we report the results from all the evaluated models in order to demonstrate the process of investigation behind the best results, rather than reporting only the best results obtained. The findings indicate that ML effectively categorizes sleep quality based on HRV and skin temperature. The classification performance of the several ML techniques investigated is shown in Table 1.

**Figure 2:**
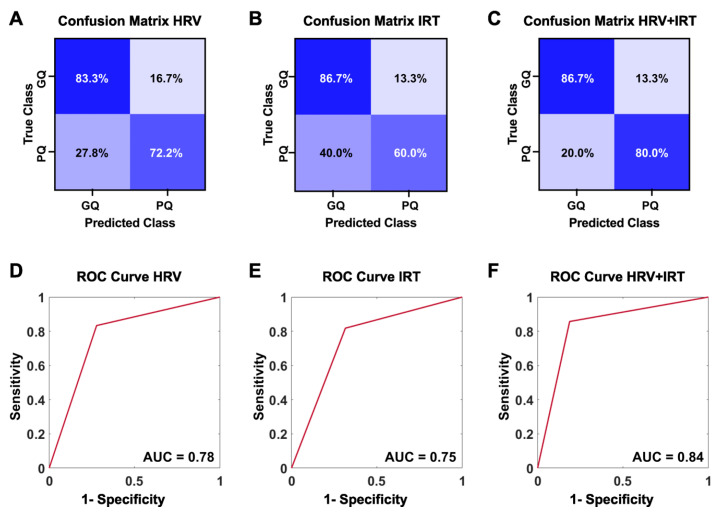
Confusion matrix graph visually representing the number of times the SVM algorithm correctly predicted the good-quality and poor-quality sleep groups using (**A**) HRV, (**B**) IRT, and (**C**) combined HRV and IRT metrics. Receiver operating characteristic curve graphs with computed area under the curve (AUC) show the difference performance obtained using (**D**) HRV, (**E**) IRT, or (**F**) combined HRV and IRT metrics as predictors.

The best classification performance was obtained by the SVM algorithms. In detail, results showed that using HRV metrics as predictors, the SVM classified sleep quality with a true positive rate (TPR) of 83.3% and a true negative rate (TNR) of 72.2% (Figure 2A). Similarly, using IRT features as predictor variables, SQ was predicted with a TPR of 86.7%, while only a TNR of 60.0% was seen (Figure 2B). Interestingly, when HRV and IRT were used in combination, the ML classification showed improved results. Indeed, sleep quality was classified with a TPR of 86.7% and a TNR of 80.0% (i.e., the highest among all the models) (Figure 2C). In summary, ROC curves showed an AUC of 0.78 for HRV metrics as predictors (Figure 2D) and a slightly lower value for IRT (0.75) (Figure 2E). Of note, the highest AUC value, 0.84, was obtained when HRV and IRT were combined for the classification (Figure 2F).

### 2.3. HRV Metrics Are Useful for Discriminating between Good and Poor Sleep Quality

**Figure 3:**
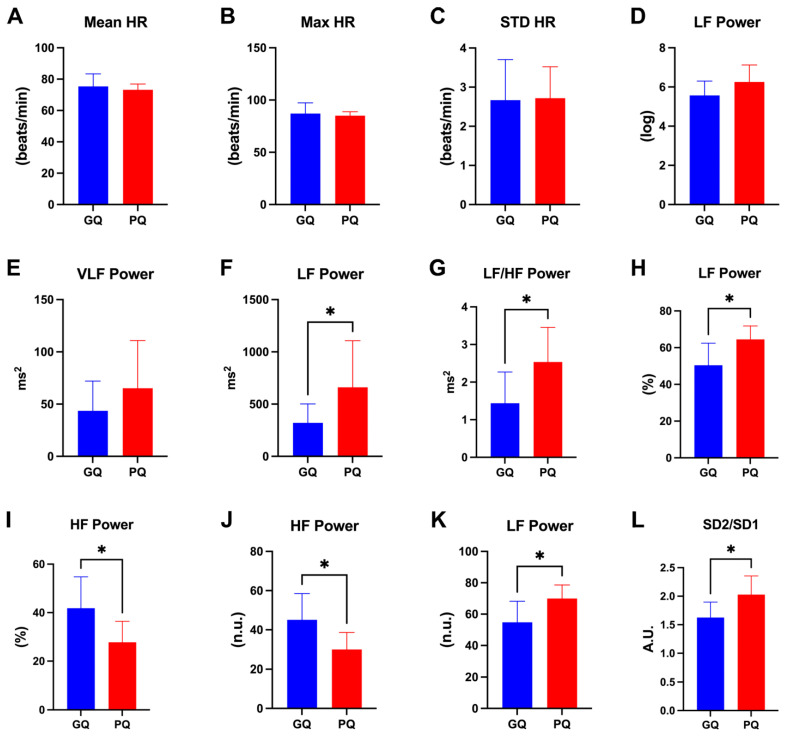
Histograms report HRV metrics in subjects with good and poor sleep quality as assessed by PSQI. (**A**) Mean heart rate; (**B**) Maximum heart rate; (**C**) Standard deviation of the heart rate; (**D**) Low-Frequency power expressed as log; (**E**) Very Low-Frequency power expressed as ms^2^; (**F**) Low-Frequency power expressed as ms^2^; (**G**) Low-Frequency/High-Frequency ratio expressed as ms^2^; (**H**) Low-Frequency power expressed as percentage; (**I**) High-Frequency power expressed as percentage; (**J**) High-Frequency power expressed as normalized units; (**K**) Low-Frequency power expressed as normalized units; (**L**) Standard deviation 2/Standard deviation 1 ratio of the Poincaré plot expressed as arbitrary units. An unpaired *t*-test was used to check for statistical differences. Data are reported as the mean and standard deviation. * *p* < 0.05, VLF = very low frequency; LF = low frequency; LF/HF = ratio between low frequency and high frequency; GQ = good quality; PQ = poor quality.

We performed an unpaired *t*-test for the selected HRV metrics used for the classification of PSQI classes (i.e., good, and poor SQ) to investigate which of them were representative of the two different classes. Regarding time-domain variables, mean HR (*p* = 0.614), max HR (*p* = 0.698), and standard deviation of HR (*p* = 0.929), no significant differences were found between subjects with good and poor SQ (Figure 3A, Figure 3B and Figure 3C, respectively). Similarly, the logarithm of LF power and raw VLF power were not statistically different between good and poor sleepers (Figure 3D and Figure 3E, respectively). On the other hand, LF (*p* = 0.011) and LF/HF power (*p* = 0.024) measured as ms^2^ showed significant differences (Figure 3F and Figure 3G, respectively). Moreover, LF power and HF power expressed as percentages (*p* = 0.034, *p* = 0.049) and normalized units (*p* = 0.040, *p* = 0.040) were different for the two classes of subjects. Finally, the SD2/SD1 (*p* = 0.013) calculated from the Poincaré plot was different between good and poor sleepers (Figure 3L).

### 2.4. Specific IRT Features Are Representative of Good and Poor Sleep Quality

**Figure 4:**
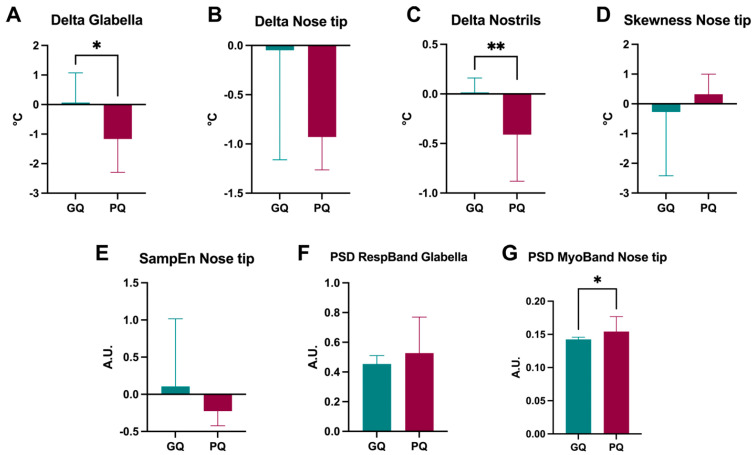
Histograms report IRT metrics in subjects with good and poor sleep quality as assessed by the PSQI. (**A**) Delta of temperature at the glabella; (**B**) Delta of temperature at the nose tip; (**C**) Delta of temperature at the nostrils; (**D**) Skewness of temperature at the nose tip; (**E**) Sample entropy at the nose tip; (**F**) Power spectrum density of the respiratory band at the glabella; (**G**) Power spectrum density of the myogenic band at the nose tip. An unpaired *t*-test was used to check for statistical differences. Data are reported as the mean and standard deviation. * *p* < 0.05, ** *p* < 0.01. SampEn = sample entropy; PSD = power spectrum density; RespBand = respiratory band; MyoBand = myogenic band.

Furthermore, to check whether the selected IRT metrics that were implemented for the classification of PSQI classes (i.e., good and poor sleep quality) were representative of good and poor sleepers, an unpaired *t*-test was used. The results highlighted that the delta values of the temperatures recorded at the glabella (*p* = 0.042) and the nostrils (*p* = 0.003) were significantly different between good and poor sleepers (Figure 4A and Figure 4C, respectively). On the other hand, the delta of the temperature of the nostrils did not show a statistical difference (*p* = 0.139), although such a result could be due to the high standard deviation found in the good-sleep-quality group (Figure 4B). Similarly, neither the temperature skewness (*p* = 0.595) nor the nose tip sample entropy (*p* = 0.485) was statistically different between the two groups (Figure 4D and Figure 4E, respectively). Additionally, the power spectrum density of the respiratory band of the glabella (*p* = 0.230) did not show differences between good and poor sleepers (Figure 4F). Finally, the power spectrum density of the myogenic band of the nose tip (*p* = 0.030) showed a significant difference between subjects with good and poor sleep quality (Figure 4G).

## 3. Discussion

The present study reports the feasibility of estimating sleep quality through an ML approach applied to HRV and skin temperature assessed through PPG and IRT, respectively. The results demonstrated good accuracy in the classification of sleep conditions using data collected during wakefulness, reaching an accuracy of 76.7% employing only HRV metrics, 73.3% when considering only thermal features, and 83.3% when merging the HRV and thermal information. Importantly, the implemented feature selection allowed the removal of redundant and useless information among the features, guaranteeing the reliable and unbiased classification performance of the classifiers. Notably, although an improvement in the classification performance was assessed when using both thermography and HRV, this improvement was not statistically significant. Moreover, we identified important metrics (for both HRV and IRT) that were significantly different between good and poor sleepers, being important in the classification of sleep quality. Importantly, the outcomes of our study are consistent with prior research. For example, Werner et al. demonstrated that individuals with elevated levels of HF of HRV measured during wakefulness (HF-HRV wake) exhibited reduced sleep latency and fewer arousals. Specifically, HF-HRV wake showed a significant correlation of −0.39 with PSQI (total score), as well as a correlation of −0.43 with sleep latency [23]. Notably, Guo et al. [24] showed that there was not a significant relationship between 24 h HRV indices and PSQI global scores. However, sleep disturbance as assessed by PSQI exhibited significant negative correlations with SDNN and LF in the waking period (r = −0.285 and −0.235, respectively). Furthermore, in some studies, higher HRV during resting wakefulness has been associated with higher actigraphy-based assessments of sleep efficiency and sleep duration in patients affected by atherosclerosis [25] and children with respiratory sinus arrhythmia [26]. Finally, Moebus and Holz [27] proposed an ML-based method for a two-class (i.e., poor and good quality) perceived sleep quality classification based on HRV, electrodermal activity, accelerometry, and skin temperature, merging information from sleeping and waking states across 30 days, achieving an accuracy of 70%.

### 3.1. HRV Metrics and Sleep Quality

The relationship between HRV during sleep and SQ has been widely investigated in the literature. For instance, Penzel et al. emphasized the correlation between sleep phases and the fluctuation of HR and HRV, specifically highlighting the impact of sleep disorders on their typical variability [28]. Carneiro and colleagues sought to examine the correlation among clinical and laboratory factors, HRV, and sleep quality in hemodialysis patients, suggesting a possible connection between HRV and sleep quality [29]. Additionally, Montesinos and co-workers demonstrated that individuals with differences in SQ had increased sympathetic activity, as shown by reduced HRV throughout the sleep period [30]. Yuda et al. investigated the correlation between the subjective evaluation of sleep quality and HRV during sleep [31]. All these studies indicate a possible connection between subjective SQ and HRV.

However, measuring HRV during sleep can pose challenges due to potential discomfort and interference with natural sleep patterns. Sleep is a critical physiological process, and introducing external monitoring devices might disrupt the individual’s ability to achieve restful sleep [32]. The discomfort associated with wearing monitoring equipment, including sensors and electrodes, could lead to altered sleep quality and impact the reliability of collected data. Consequently, HRV measurements during wakefulness are often preferred as they allow, for more comfortable and non-intrusive monitoring [33]. Awake HRV assessments can be conducted without disturbing the natural sleep environment, enabling individuals to maintain their regular sleep routines [34]. Additionally, awake measurements provide insights into the baseline ANS activity and can be easily integrated into daily activities, offering a more practical and convenient approach for longitudinal studies and continuous monitoring, particularly in clinical or real-world settings.

In the present study, measuring HRV during wakefulness resulted in good accuracy in the prediction of SQ. Moreover, several variables were found to be representative of good and poor sleepers. For example, the poor sleepers showed a higher LF and LF/HF than people with good SQ. Several studies have demonstrated a negative correlation between SQ and the LF component of HRV as well as LF/HF. For instance, Hsu et al. found negative correlations between SQ and HRV, including total power, LF, and LF/HF [35]. Furthermore, Tobaldini and colleagues reported a significant increase in the LF component of HRV in insomniacs compared to healthy subjects during sleep, suggesting a predominant sympathetic modulation in insomnia across sleep stages [36]. These findings suggest that LF of HRV is associated with poor SQ in the normal population, and it is in accord with our results.

On the other hand, we report that HF was higher in the subjects with good SQ. Studies have shown that the normalized spectral HRV measures, particularly the HF component, are frequently used to quantify the modulation of the parasympathetic branch of the ANS during sleep [37]. In this regard, Fatt and co-workers indicated that higher HF is associated with better SQ, and people with chronic fatigue syndrome show statistically lower HF than a control population [38]. The fact that we also found a difference in the HF of HRV between healthy subjects with good and poor SQ corroborates the sensitivity of this metric in discriminating SQ with data collected during wakefulness.

In the context of HRV analysis, the Poincaré plot is used to calculate SD1 and SD2, which are important measures reflecting short-term and long-term HRV, respectively [39]. SD1 is associated with parasympathetic modulation, while SD2 reflects sympathetic activity [39]. Additionally, SD1 correlates with baroreflex sensitivity and HF power, which are indicative of the change in interbeat interval duration per unit change in blood pressure and parasympathetic activity, respectively [39]. On the other hand, SD2 represents both short-term and long-term HRV and is associated with sympathetic activity [39]. Additionally, the ratio between SD2 and SD1 (SD2/SD1) measures the unpredictability of the RR series. Interestingly, in the present investigation, SD2/SD1 showed statistical differences between the subjects with good and poor SQ. This result highlights SD2/SD1 as an important variable for recognizing the quality of sleep while awake.

### 3.2. Skin Temperature Measured by IRT and Sleep Quality

The correlation between skin temperature and sleep quality has been the object of interest in various studies. Raymann and colleagues demonstrated a correlation between skin temperature and sleep-onset latency [40]. Furthermore, changes in skin temperature could affect sleep, indicating a potential bidirectional relationship between the two variables [41]. Additionally, Ko et al., found a weaker correlation between core temperature and sleep propensity compared to the correlation between distal or proximal skin temperature and sleep onset, suggesting that skin temperature may play a more significant role in sleep initiation [42]. During sleep, there is a relative vasodilation of distal skin compared with proximal skin, leading to a reduction in the distal–proximal skin temperature gradient [43]. In this perspective, Romeijn provided an overview of the neuroanatomical pathways and physiological mechanisms by which skin temperature can affect the regulation of sleep and vigilance [44]. Moreover, it was demonstrated that the skin temperature of subjects was more evenly distributed across the body surface during sleep than during wakefulness [45]. Additionally, van der Heide et al. highlighted the association between skin temperature and sleep, stating that wake is associated with a relatively low skin temperature and a relatively high core body temperature, while sleep is associated with a higher skin temperature and a lower core body temperature [46]. Furthermore, it was found that periocular skin warming promoted sleep onset, indicating a potential therapeutic approach to improving sleep quality through skin temperature manipulation [47]. These findings collectively suggest a strong correlation between skin temperature and SQ. Despite the intricate interplay between body temperature regulation and sleep, there are no studies that have thoroughly investigated the relationship between skin temperature during wakefulness and sleep quality. Importantly, one of the novel aspects of this study relies on the assessment of the relationship between facial skin temperature during wakefulness and SQ, demonstrating the possibility of using ML algorithms to accurately classify SQ from IRT measurements.

### 3.3. Practical Implications

The correlations between HRV, skin temperature, and SQ have profound implications for ergonomic design in workplace settings. By integrating HRV and IRT monitoring into workplace health programs, employers can assess an employee’s SQ indirectly and non-invasively in an objective manner. This enables the implementation of personalized ergonomic solutions, such as adjusting work schedules, optimizing office lighting and temperature, or recommending breaks to mitigate fatigue. Consequently, such measures could lead to enhanced cognitive performance, increased productivity, and reduced risk of errors or accidents due to sleep-related impairments. However, it should be highlighted that integrating wearable technology in the workplace for sleep assessment necessitates robust data protection measures to safeguard individuals’ privacy rights. Ensuring the use of data encryption, secure data storage, and user consent mechanisms is essential to mitigate privacy risks associated with using wearable devices in working contexts [48].

The feasibility of using these measurements during wakefulness opens avenues for real-time health monitoring. For instance, wearable technology incorporating HRV analysis could alert users to deteriorations in SQ, prompting timely interventions such as stress management techniques or lifestyle adjustments. This proactive approach to monitoring and addressing SQ issues could play a significant role in preventing chronic health conditions associated with poor sleep, such as cardiovascular diseases and mental health disorders.

Our research contributes to the broader field of sleep study, providing a novel, non-invasive methodology for SQ assessment. This methodological advancement could encourage further research exploring the interplay between daytime physiological markers and various aspects of sleep, potentially unveiling more intricate connections and leading to refined assessment tools.

Beyond ergonomic and personal health applications, this research could have implications in fields such as sports science, where athletes’ sleep quality is crucial for performance and recovery; advanced driver assistance systems (ADAS), for driver drowsiness assessment; education, to monitor and improve students’ SQ; and military applications, where these methods could be used to monitor soldiers’ readiness and mitigate the risks associated with sleep deprivation, such as impaired judgment or decreased combat performance.

Concerning the costs and work needed to implement such a solution in a real context, it should be considered that HRV could be easily obtained from a wearable sensor (e.g., smartwatches) and that facial temperature could be measured through a low-cost thermal camera, hence resulting in inexpensive and affordable solutions.

### 3.4. Strengths and Limitations

The strengths and limitations of this study should be considered. For example, gender unevenness arose due to higher female participation in the work setting where recruitment took place, reflecting broader trends of greater female engagement in health-related activities. However, in the present study, gender imbalance does not affect the results because we predicted the SQ for each individual subject, and the objective was not to compare the two unbalanced groups. In addition, such an imbalance does not represent an important limiting factor in this case since the strict cross-validation used ensures the good generalizability of the results. Nevertheless, further studies involving larger samples would certainly be beneficial to confirm the findings. Additionally, we know that SQ varies with age, and we are aware that the further studies needed to generalize across multiple ages and models across different ages would be valuable. We focused on this age group because at this age it is very important to evaluate the SQ, given the incidence of sleep disorders increases around the age of 50 [49,50]. Additionally, it is crucial to highlight that evaluating SQ during the awake state could be beneficial for early detection of sleep disorders, continuous and non-intrusive monitoring, and informed lifestyle adjustments to improve sleep hygiene. In this perspective, the development of such models can enhance cognitive function, mood, and daytime performance, providing an important tool for managing mental health conditions linked to sleep disturbances, hence improving overall health. Additionally, it ensures safety in critical occupations (e.g., drivers and pilots) by preventing accidents due to poor sleep. Finally, the implementation of models as those proposed in this study can allow for large-scale data collection, aiding research and public health initiatives based on artificial intelligence tools, contributing to a better understanding of sleep patterns and the development of effective interventions.

### 3.5. Important Remarks

Importantly, to quantitatively assess the quality of the contribution of the findings, several performance indicators employed and highlighted in this study must be considered:

(i) Accuracy of Classification: The overall accuracy of the metrics for classifying sleep conditions was 76.7% for HRV metrics, 73.3% for thermal features, and 83.3% for combined HRV and thermal information.
(ii) Feature Importance: Key HRV metrics such as LF, LF/HF, and HF were identified as significant contributors to the classification performance.
(iii) Poincaré Plot Analysis: Significant differences in the SD2/SD1 ratio were observed between good and poor sleepers, indicating its potential as a reliable indicator of sleep quality.
(iv) Receiver Operating Characteristic (ROC) Curve: The ROC curve analysis for the combined model yielded an area under the curve (AUC) of 0.88, indicating high discriminative ability.
(v) Cost-Effectiveness: The study highlighted the affordability of implementing the proposed solution using wearable sensors and low-cost thermal cameras, emphasizing its practical applicability in real-world settings.

## 4. Materials and Methods

### 4.1. Experimental Procedure and Data Acquisition

The study comprised a sample of 28 individuals who were in good health (no chronic diseases, such as cardiovascular diseases, diabetes, or chronic respiratory conditions; no acute illnesses or infections; non-smoking; no diagnosed psychological/psychiatric conditions), consisting of 20 female and 8 male participants, with an average age of 51.46 ± 7.68 years. The participants were instructed to abstain from engaging in intense physical activity and to avoid alcohol and caffeine for at least 48 h before the measurements. Additionally, they were also instructed to avoid using moisturizing cream and make-up, which can impair thermal measurements. The participants were instructed to comfortably lie on a medical cot and rest. The duration of the experimental session was 5 min. This specific time window was chosen based on established research and methodological considerations in the field of HRV measurement [51,52].

To gather data on the pulse rate variability of the subjects, a PPG sensor manufactured by HeartMath, Inc. (emWave Pro Plus) was utilized. The sensor was positioned on the left-hand index fingertip of each participant during the task. The sampling frequency utilized was 370 Hz.

Simultaneously, a digital thermal infrared camera, the FLIR SC660 (FLIR, Wilsonville, OR, USA), was utilized to measure the facial temperature. The camera features a 640 × 480 bolometer FPA, with a sensitivity/noise-equivalent temperature difference of <30 mK at 30 °C, and a field of view of 24° × 18°. The IRT device was positioned at a distance of 60 cm from the participant and directed towards the facial region. The frequency of sampling utilized was 10 Hz. The camera underwent blackbody calibration to mitigate any potential sensor response drift or shift and optical anomalies. The thermal imaging acquisitions were conducted following the standard guidelines [53]. The experiment was conducted in a thermoneutral environment to mitigate the potential impact of thermoregulatory-induced alterations. Additionally, the subjects were given 15 min to acclimate to the environment before the session to attain thermal equilibrium [53]. Additionally, it is noteworthy that all sessions were arranged to take place at a consistent time of day, to mitigate the potential impact of any circadian rhythm fluctuations [54]. We conducted the measurements between 10 and 11 a.m. This specific time window was chosen for several reasons. First, by 10 a.m., all participants had completed their morning routines, including breakfast, and were in a stable physiological state. This reduces variability that might be present immediately after waking or during the early morning transition period. Additionally, conducting the measurements before noon helps avoid the post-lunch dip in alertness and physiological changes that occur after eating, which can affect HRV and skin temperature.

The research was approved by the Research Ethics Board of the University of Chieti-Pescara, with an assigned approval number of 1479 and a date of approval of 5 March 2017. The study adhered to the principles outlined in the Declaration of Helsinki. All participants provided their informed consent and were given the option to withdraw from the experiment at any point.

### 4.2. Data Preprocessing

Regarding PPG, the signals underwent band-pass filtration with the cutoff frequencies set at 0.2 and 10 Hz. The PPG signals that have been filtered and normalized (z-score) are subjected to an automated peak identification procedure. The algorithm’s performance was evaluated through visual inspection, and no corrective measures were deemed necessary. The peaks of the PPG signal were utilized to assess the HRV metrics during the one-minute recording. The study involved the computation of several metrics, including both time-domain (e.g., heart rate, HR), and frequency-domain features (e.g., the low-frequency (LF) and high-frequency (HF) power of HRV and their ratio, LF/HF). Such metrics were extrapolated using Kubios HRV Standard 3.4.0 software as previously reported [21,55,56].

The IRT recordings underwent a quality assessment through visual inspection, and no video recordings were deemed unsatisfactory. Three regions of interest (ROIs) were chosen on the facial area, namely, the glabella (G), the tip of the nose (NT), and the nostrils (N). A tracking algorithm was employed to follow the position of the ROIs through the frames of the video recordings [57]. Due to the participants’ minimal movement throughout the experiment, the algorithm successfully tracked all frames without any failures. The temperature time course of each ROI was analyzed to extract the relevant features for input into the computational models. The following parameters were computed for the signal: temperature variation (ΔT), mean value, standard deviation (SD), kurtosis, skewness, sample entropy (SampEn), 75th percentile, and power spectral density (PSD) for the respiratory (PSD-breath), cardiac (PSD-cardiac), and myogenic (PSD-myo) frequency bands. Specifically, ΔT was determined by calculating the difference between the averages of the first and last 5 s of the acquisition, providing information regarding the signal variability. The various moments of the temperature distribution, such as mean value, standard deviation, kurtosis, and skewness, were evaluated to provide insights into the central tendency, dispersion, and shape of the temperature’s temporal evolution [58]. SampEn is mathematically expressed as the negative natural logarithm of the conditional probability and assesses the nonlinear predictability of the signal. This probability is based on the matching of signal subseries of a specific length, referred to as pattern length, within a given tolerance range, known as the similarity factor [59]. The PSD characterizes the allocation of power among the various frequency constituents that constitute a given signal. In this study, the mean PSD across specific frequency bands, namely, the myogenic band (0.04–0.15 Hz), respiratory band (0.15–0.5 Hz), and cardiac band (0.5–1 Hz), was computed [60]. It is noteworthy that prior to being utilized in the ML framework, all features underwent normalization (z-scores).

### 4.3. Statistical Analysis

The 2-class classification of the quality of sleep (good and poor SQ, with PSQI = 5 as cut-off score) from HRV and IRT features, both separately and together, were performed through Decision Tree (DT), linear-kernel Support Vector Machine (SVM), k-nearest neighbor (KNN), Ensemble (ENS), and neural network (NN) models. The two classes were balanced (13 participants had poor SQ, and 15 exhibited good quality of sleep, resulting in 28 samples for the models), and to prevent overfitting effects and evaluate the models’ ability to generalize, a 5-fold cross-validation approach was employed. It is noteworthy that a subset of the features was selected using the wrapper method [61] and used as input for the ML framework. The wrapper method for feature selection is a search algorithm that aims to identify the most relevant subset of features for a given target variable. This method explores all possible combinations of features and evaluates their performance using a specific metric. The search process continues until a stopping criterion is met, such as a limited number of iterations or no further improvement in performance. In this study, a sequential forward selection approach was used, where features are added one by one based on their ability to improve the model’s performance. To evaluate the performance of the classifier, the confusion matrix was computed, providing the sensitivity, specificity, and accuracy of the procedure. Furthermore, a receiver operating characteristic (ROC) analysis was performed, and the area under the ROC curve (AUC) was computed. The metrics selected by the wrapper procedure were investigated through an independent-samples *t*-test to assess differences between the groups with good and poor quality of sleep.

The MATLAB 2023b software (MathWorks, Inc., Natick, MA, USA) was utilized for conducting data preprocessing and analysis, while GraphPad Prism version 10.1.1 (Boston, MA, USA) was used to make graphs. 

## 5. Conclusions

This research examines the possibility of using ML techniques to evaluate SQ by analyzing HRV and IRT data while the individual is awake. The findings indicated that it is feasible to assess SQ with an accuracy of 83.4% by analyzing multimodal HRV and IRT signals using SVM. These findings provide opportunities for novel ergonomic applications that can monitor SQ in workers, students, and athletes without the need for intrusive methods. Achieving an accuracy of 83.4% demonstrates the potential of multimodal HRV and IRT signals to predict sleep quality effectively. This level of accuracy is significant, considering the non-intrusive nature of the measurement techniques. In addition, the integration of HRV and IRT for SQ assessment is a novel approach that contributes to the existing body of knowledge. Indeed, this multimodal method shows promise for developing wearable and contactless devices that can be used in real-world settings. Nevertheless, the sample size was relatively small and may not fully represent the diversity of the general population. Future research should include a larger and more varied sample to validate the findings across different demographics and lifestyle factors. Additionally, conducting longitudinal studies to track changes in SQ over time and their relationship with HRV and IRT metrics could provide deeper insights into the temporal dynamics of sleep health. In addition, combining HRV and IRT with other physiological and behavioral biomarkers could enhance the robustness and accuracy of SQ predictions. Developing and testing wearable devices and contactless systems that utilize HRV and IRT for sleep quality assessment in real-world settings, such as workplaces, schools, and athletic environments, is a crucial next step. In conclusion, this study demonstrates the feasibility of using HRV and IRT data analyzed with ML techniques to assess SQ in a non-intrusive manner. While the results are promising, further research is needed to address the limitations and expand the applicability of this approach. The potential for developing practical and effective tools for monitoring and improving SQ in various populations remains a promising avenue for future exploration.

## Figures and Tables

**Table 1:** Machine learning models’ performance expressed as the true positive rate (TPR), true negative rate (TNR), and accuracy for the different feature sets considered (i.e., HRV, IRT, and HRV + IRT).

Feature Set	Model	TPR	TNR	Accuracy
HRV	DT	66.7	66.7	66.7
	SVM	83.3	72.2	77.8
	KNN	80.0	40.0	60.0
	ENS	73.3	73.3	73.3
	NN	86.7	46.7	66.7
IRT	DT	73.3	66.7	70.0
	SVM	86.7	60.0	73.4
	KNN	80.0	46.7	63.4
	ENS	86.7	60.0	73.4
	NN	66.7	66.7	66.7
HRV + IRT	DT	80.0	26.7	53.4
	SVM	86.7	80.0	83.4
	KNN	80.0	46.7	63.4
	ENS	80.0	53.3	66.7
	NN	86.7	60.0	73.4

## Data Availability

The data presented in this study are available on reasonable request from the authors.
